# Nutritional strategies for childhood obesity treatment and prevention without counting calories—A narrative review

**DOI:** 10.1002/pdi3.2524

**Published:** 2025-01-10

**Authors:** Karolina Kuźbicka

**Affiliations:** ^1^ Department of Pharmacology Medical University of Gdańsk Gdańsk Poland

**Keywords:** childhood obesity, eating habits, emotional eating, healthy eating, obesity treatment

## Abstract

Treating obesity in children is a significant challenge for modern medicine and a delicate matter at the same time. Although obesity treatment typically involves lifestyle modifications; in particular implementation of a detailed menu and calorie restrictions, when it comes to children, imposing a specific menu can be tough and consequently counterproductive. Therefore, it is advisable to introduce some small single steps and simple rules to lower the risk of obesity without causing unnecessary pressure. This article includes practical advice on how to treat and prevent excessive body weight in children. While primarily aimed at professionals working with children and their parents or guardians—such as teachers, dietitians, and pediatricians—these recommendations can also be adapted for parents and caregivers in various formats to effectively reach and benefit this target group.

## 1 INTRODUCTION

Obesity among children presents a growing challenge for contemporary medicine. Constraints imposed by the COVID‐19 pandemic have particularly exacerbated this issue among school‐age children and adolescents.^1^ Excess body mass not only poses numerous physical and mental health problems during childhood but also elevates the risk of obesity in adulthood, thereby shortening life expectancy.^2^ Obesity is a complex multifactorial disease influenced by a combination of genetic, environmental, behavioral, and metabolic factors. Initial treatment of obesity typically involves lifestyle modifications, in particular implementation of a detailed menu and calorie restrictions. However, when working with overweight children, imposing a specific menu can be challenging as children often prefer some autonomy in their food choices. Therefore, it may not only be ineffective but could also be counterproductive to strictly enforce a meticulously planned menu. According to Shunk et al., excessive focus on weight control and strict dietary limitations among young girls at risk for overweight were associated with bigger weight gain between the age of 5 and 9.^3^ Hence, it is crucial to find ways to improve a child's diet and reduce the risk of obesity but without subjecting them to excessive pressure and counting calories as it can generate unnecessary stress for both the child and his parents. The article provides some advice aimed at altering a child's menu to enhance his nutrition status and decrease the risk of overweight. While primarily aimed at professionals working with children and their parents or guardians—such as teachers, dietitians, and pediatricians—these recommendations can also be adapted for parents and caregivers in various formats to effectively reach and benefit this target group.

## 2 SEARCH STRATEGY

The idea of this article was created as a result of the collected experience in working with overweight and obese children and their families. The articles cited in this narrative review were obtained by an electronic search of the PubMed and Scopus databases for literature published from 2004 to 2024 in English. The search strategy employed a range of keywords, including but not limited to: “children's obesity,” “eating habits,” “breakfast AND obesity,” “snacks AND obesity,” “baby‐led weaning (BLW) method,” and “emotionally eating.” A preliminary screening of each article's title and abstract was followed by a full‐text assessment to include relevant studies.

## 3 DISCUSSION

### 3.1 Eating only during meals

It is essential to have a fixed number of planned meals throughout the day, ideally with stable mealtimes that can be adjusted by up to 30 min based on circumstances. If dinner cannot be eaten at its usual time, another, probably smaller meal can be consumed then with dinner postponed to a later time such as supper. This approach encourages the consumption of more nutritious foods and discourages snacking between meals. Establishing regular meal patterns has additional benefits, such as improving fasting lipid and postprandial insulin profiles and thermogenesis.^4^ The earlier regular mealtimes are established, the easier it becomes to maintain them during adolescence and adulthood. Children should avoid eating or drinking any sugar containing beverages between planned meals to maintain routine and regulate appetite. It is worth noting that sugary drinks are considered snacks as they elevate the serum sugar level. It was showed that high frequency of snacks, but not high frequency of meals, is associated with a greater risk of overweight and abdominal obesity in US children, with “meal” defined as a planned meal and “snack” as a spontaneous one.^5^ Unplanned snacks significantly contribute to daily caloric intake and are a significant factor in obesity development. O’Kane et al. evaluated 21 articles and found that snacks provided 231–565 kcal daily, which can constitute up to one‐third of a child's daily caloric demand.^6^ It is important not to keep food in children's rooms or other accessible areas at home, as this encourages eating for reasons other than hunger, such as boredom or anxiety. Eating in response to emotional states is a significant risk factor for obesity.^7^ Eating meals should be a conscious celebratory activity not merely an addition to playtime or doing other activities. Decreasing the availability of salty snacks was shown to reduce their consumption among Norwegian children.^8^ Furthermore, it is highly beneficial for a children to have as many meals as possible together with the family, as this reduces the risk of obesity and promotes the consumption of healthy foods.^9^

### 3.2 Eating early meals

Breakfast is often hailed as the most important meal of the day. Nevertheless, it is frequently skipped in the United States and Europe, with rates ranging from 10% to 30%, depending on the age group and population.^10^ Research shows that children who eat breakfast are less likely to be overweight,^11^ have superior nutritional profiles, and demonstrate improved cognitive function.^10^ Skipping breakfast is also associated with the consumption of energy‐dense carbohydrate‐rich snacks in the afternoon and evening.^12^ It is not just the presence of breakfast that matters, and its quality is equally important. Studies suggest that dietary fiber plays a crucial role in regulating appetite and increasing satiety,^13^ which helps to plan wisely the subsequent meals of the day. Breakfast products rich in dietary fiber include whole‐grain cereals, whole‐grain bread, nuts, seeds, fruits, and vegetables. Additionally, dairy products such as milk, yogurt, and white cheese are vital elements of breakfast, providing calcium and vitamin D3, which is crucial for bone and teeth health. Two systematic reviews by Gimenez‐Lagarre et al. confirmed that children who usually ate breakfast had higher fiber intake, consume more fruits and vegetables, and had significantly higher daily calcium intake compared to those who skipped breakfast.^14^, ^15^ However, many breakfast products commonly consumed by children, such as sweetened breakfast cereals, white bread, biscuits, and cakes, are high in free sugars.^16^ A diet abundant in free sugars can contribute to obesity and a lack of dietary diversity, leading to deficiencies in vitamins and minerals.^17^ Encouraging healthier breakfast choices can be achieved through small single steps such as serving porridge with melted milk chocolate instead of chocolate breakfast cereals or offering natural yogurt with dried fruits and seeds instead of sweetened homogenized cheese or fruit yogurt. Once such innovations are accepted, parents can introduce further changes to promote healthier eating habits.

### 3.3 Simple proportions of products' groups, not specific products

Treatment of obesity in adults typically involves focusing on reducing overall caloric intake.^18^ However, when addressing childhood obesity, it is often more effective and less stressful for both children and parents to establish some simple rules rather than meticulously planning meals and counting calories throughout the day. One approach is to adopt a plate‐based method according to which the recommended proportions of three food groups are visually represented on a plate, without specifying daily servings or portion sizes.^19^ However, it is important to note that this approach may not ensure that children consume all the foods served, especially with the growing popularity of advised by the pediatrics BLW method, which allows children to choose freely from the plate.^20^

Another strategy is to focus on the number of portions from various food groups.^19^ For instance, adults can supervise that children consume three portions of accepted vegetables, one portion of accepted fruits, two portions of carbohydrates (such as whole‐grain bread or grains), and a maximum of one portion of sweets. Portion sizes should be determined depending on the child's age and nutrition requirements. Older children and adolescents should not only be involved in establishing these rules but also take responsibility for adhering to them. Whenever possible, they should also participate in shopping for and preparing meals. By incorporating fixed portions of low calorie‐dense foods, it is likely that the total daily calorie intake will decrease.

Additionally, there is evidence suggesting that the size of the dish used for serving food influences the amount of food that is self‐served,^21^ indicating that using smaller dishes could help reduce calorie intake. However, some studies contradict this, suggesting that plate or bowl size may not significantly impact the amount of food consumed.^22^

Using straightforward instructions like focusing on the proportions of different food groups or adopting a plate‐based approach without detailed food itemization and calories counting tend to be less complex and more flexible.^23^ These approaches offer a better chance for long‐term changes in nutrition, which are crucial for a child's dietary habits into adulthood.

### 3.4 Attitude towards food more important than vegetables and calorie counting

When children's nutrition is considered, it is crucial to prioritize their attitude towards food over short‐term changes in quantity or quality of their diet. Developing healthy eating habits and a positive relationship with food during childhood significantly reduces the risk of obesity in the future.^24^ Children should not be coerced or manipulated into eating specific food considered “healthy.” It was showed that parents who employ restrictive feeding practices for weight control increase the risk of their preschool children developing overweight or obesity by 1.75 times compared to those who do not use such practices.^25^ The most effective way to encourage children to eat healthy foods is by demonstrating that these foods are willingly be eaten by other family members. It was proven that the eating behaviors of parents had a significant impact on the nutrient intake of their preschool children.^26^

Sometimes parents attribute the lack of dietary variety in their children's diets to food neophobia or “picky eating.”^27^ It is worth to remember that offering a new food may result in initial refusal from the child. However, consistently offering new products, especially when the entire family eats those, increases the likelihood of children trying and eventually accepting these products.^28^ While raw vegetables are often recommended, they can also be served as pickled or cooked to provide more options. Parents should be aware that the atmosphere surrounding mealtime is just as important as the meals themselves. Moreover, various motivating techniques can be employed, depending on the child's age. For example, creating a poster where children can mark or place stickers after completing a portion from a specific food group or trying a new food can be very rewarding and promote dietary diversity. For older children, involving them in menu planning and cooking can further enhance their interest and investment in healthy eating habits.

## 4 CONCLUSIONS AND IMPLICATIONS FOR PRACTICE

Obesity is an increasingly prevalent issue among children and teenagers. To treat or prevent excessive body weight effectively, it is crucial to take a comprehensive approach that considers the child's diet, physical activity, and lifestyle. As far as diet is concerned, relying on restrictive menus and calorie counting can be stressful and exhausting. Instead, it is better to implement gradual changes to eating habits and adhere to a few simple dietary rules, without exerting unnecessary pressure. A limitation of this review is that it focuses solely on nutritional interventions, without considering the role of physical exercise and psychological support.

Advice for professionals working with children and their parents or guardians:

‐ Fixed mealtimes should be established. Even if dinner cannot be served at the usual time, an alternative meal should be offered and dinner postponed to a later time, such as supper time.
‐ All family members should be discouraged from eating and drinking sweetened beverages between scheduled mealtimes. Keeping snacks or fruits within the child's sight should be avoided.
‐ As many meals as possible should be eaten together by the whole family.
‐ Breakfast should always be served, even if it is small or contains some sugar initially. Products containing more fiber and less sugar should be incorporated slowly.
‐ Target number of daily portions from specific food groups should be established, with an emphasis on the most important groups, such as vegetables and healthy fats such as those from vegetable sources.
‐ It is important to foster a positive atmosphere when discussing food, avoiding any intimidation or coercion. The eating habits developed during childhood are crucial in shaping lifelong health.

By implementing these recommendations, both professionals working with children and parents can help foster healthier eating habits, contributing to the overall well‐being of the children both now and in the future.

## AUTHOR CONTRIBUTIONS

Karolina Kuźbicka created this article as a result of the collected experience in working with overweight and obese children and their families.

## CONFLICT OF INTEREST STATEMENT

The author declares no conflicts of interest.

## ETHICS STATEMENT

This article required neither Institutional Review Board approval nor Ethical Committee approval.

## Data Availability

The data that support the findings of this study are available from the corresponding author upon reasonable request.
